# Lack of Association of Apolipoprotein E (Apo E) ε2/ε3/ε4 Polymorphisms with Primary Open-Angle Glaucoma: A Meta-Analysis from 1916 Cases and 1756 Controls

**DOI:** 10.1371/journal.pone.0072644

**Published:** 2013-09-02

**Authors:** Wei Wang, Minwen Zhou, Wenbin Huang, Shida Chen, Xiulan Zhang

**Affiliations:** Zhongshan Ophthalmic Center, State Key Laboratory of Ophthalmology, Sun Yat-Sen University, Guangzhou, People's Republic of China; Bascom Palmer Eye Institute, University of Miami School of Medicine; United States of America

## Abstract

**Background:**

A number of case-control studies were conducted to investigate the association of apolipoprotein E (Apo E) polymorphisms with primary open angle glaucoma (POAG). But the results remain controversial. This meta-analysis aims to comprehensively evaluate the relationship between a common ε2/ε3/ε4 polymorphism in Apo E gene on the risk of POAG.

**Method:**

A comprehensive literature search for studies published up to April 2013 was performed. Summary odds ratios (ORs) and 95% confidence intervals (CI) were calculated employing random-effects models irrespective of between-study heterogeneity. Publication bias of literatures was evaluated using funnel plots and Egger's test.

**Results:**

A total of 12 studies including 1916 cases and 1756 controls meeting the predefined criteria were involved in this meta-analysis. Overall, the Apo E ε2 allele and ε4 allele were not associated with POAG, compared with those carrying ε3 allele, with ORs of 0.98 (95% CI, 0.79 to 1.23; P = 0.872) and 1.05 (95% CI, 0.78 to 1.41; P = 0.743), respectively. Genotypic analysis also found no significant association between the ε4 carriers (ε3/ε4+ε4/ε4), ε2 carriers (ε2/ε3+ε2/ε2) and POAG, compared with participants with Apo E ε3/3, with ORs of 0.91 (95% CI, 0.66 to 1.25; P = 0.543) and 1.08 (95% CI, 0.74 to 1.57; P = 0.694), respectively. In the subgroup analysis by ethnicity, source of controls, genotyping methods, Hardy-Weinberg equilibrium or not, or type of the POAG, still no obvious associations were found.

**Conclusions:**

This meta-analysis suggests that Apo E ε2/ε3/ε4 polymorphisms may not be associated with the risk of POAG. However, well-designed studies with larger sample size and more ethnic groups are required to further validate the results.

## Introduction

Glaucoma is a leading cause of irreversible blindness, estimated to affect 79.6 million people by 2020, of which over 8 million will suffer from bilateral blindness [1]. Primary open-angle glaucoma (POAG), clinically classified into high tension glaucoma (HTG) and normal tension glaucoma (NTG), is the most prevalent form of glaucoma in most populations, and affects 70 million individuals worldwide [2].

POAG is considered to be caused by multiple genetic and environmental factors, and interactions among these factors [2], [3]. Three causative genes, namely optineurin (OPTN), myocilin (MYOC), and WDR36, have been identified thus far, but these account for fewer than 10% of patients with sporadic, adult-onset POAG [2]. Quite a number of POAG susceptibility genes have been identified. One of the good potential candidate susceptibility gene had been studied is apolipoprotein E (Apo E) [4].

Apo E is the principal apolipoprotein within the central nervous system and polymorphic variants of Apo E have been associated with a number of neurodegenerative diseases, including Alzheimer's disease [5], [6]. It is a ligand for the low density lipoprotein family of receptors and plays a pivotal role in cholesterol metabolism. Apo E has three common isoforms, ε2, ε3, and ε4, respectively, at a single locus in chromosomal region 19q13.2. These alleles define six Apo E phenotypes: ε2/ε2, ε2/ε3, ε2/ε4, ε3/ε3, ε3/ε4, and ε4/ε4. Apo E ε3/ε3 is the most predominant genotype and ε3 is the most common allele in majority of populations. Individuals with one ε4 allele gene are three- to four-times more likely to develop AD than those without an ε4 allele gene [6]. The neuronal injuries associated with Alzheimer disease have several similarities with the optic nerve changes often seen with POAG [7]. Thus, the Apo E gene appears to be a potential genetic marker for POAG.

To date, many case–control studies have been carried out to investigate the role of the Apo E gene polymorphism in the development of POAG, but these have produced conflicting or inconclusive results. Some of the studies showed an association between certain types of Apo E alleles and POAG, whereas others found no association. In a study by Junemann et al [8], they found a significant association between the level of IOP and the Apo E ε2 allele, however, Mabuchi et al [9] and Yuan et al [10] found a reduction in POAG risk in people with ε2 allele in a Japanese population and Chinese population, respective. Vickers et al [11], Al-Dabbagh et al [12] and Yuan et al [10] reported that the Apo E ε4 gene is associated with elevated risk of POAG or NTG. On the contrary, Lam and his colleagues [13] reported that the Apo E ε4 gene confers a protective effect against NTG. Their findings, however, could not be replicated in other researchers [14], [15], [16], [17], [18], [19], [20]. As a result, the role of Apo E in POAG remains to be established.

To date, no meta-analysis has been conducted to evaluate the association of the polymorphisms of Apo E with POAG. Hence, we performed a meta-analysis of all eligible studies to derive a more precise estimation of the association, to help us better understand its possible influence on POAG.

## Methods

This meta-analysis was performed according to a predetermined protocol described in the following paragraph. MOOSE guidelines were followed at all stages of the process [21].

### Literature Search

The Pubmed, Embase, ISI Wed of Knowledge, Cochrane Library and Chinese databases such as the China National Knowledge Infrastructure (CNKI) and Wanfang were searched (up to April 1, 2013). The Medical Subject Terms (MeSH), keywords and free text words used for this research were apolipoprotein E or Apo E, polymorphism (s) or allele (s) variation or genotype (s) and glaucoma or intraocular hypertension. Hand-searching of the references of included articles identified was also performed to identify other relevant studies. If the overlapping patient population was included in several studies, the latest study was included. If more than one geographical or ethnic population were included in one article, each population was considered separately. Two investigators (Wang W and Zhou MW) independently screened the information including the titles, abstracts and full texts to determine inclusion carefully. If the two reviewers disagreed with each other, a third reviewer (Zhang XL) may be sought.

### Quality assessment

The qualities of included studies were assessed independently by the same two investigators using the Newcastle-Ottawa Scale (NOS) [22]. The NOS uses a ‘star’ rating system to judge quality based on 3 aspects of the study: selection, comparability, and exposure (case–control studies) or outcome (cohort studies). Scores were ranged from 0 star (worst) to 9 stars (best). Studies with a score of 7 stars or greater were considered to be of adequate quality. Disagreement was settled as described above.

### Inclusion/Exclusion Criteria

The inclusion criteria were as follows: (1) studies on the relationship between Apo E ε2/ε3/ε4 gene polymorphism and POAG; (2) case-control study using either a hospital-based or a population-based design (Studies were classified as population-based if the controls were selected from the same source population as the case-patients, including the community and the general population. Studies using hospital- or clinic-based patients with other illnesses as controls and studies that used an unidentified healthy control group were considered hospital-based.); (3) studies with full text articles; (4) sufficient data for estimating an odds ratio (OR) with 95% confidence interval (CI); and (5) not republished data. Studies were excluded if they were family studies; or published abstracts from meeting.

### Data extraction

The same two reviewers independently extracted data, cross-checked, discussed all conflict, and reached consensus on all items. Following data were extracted from each study: first author's last name, publication date, population ethnicity, study design, study location, age, sex, methods of genotyping, number of cases and controls, and available allele and genotype frequencies information.

### Statistical Analysis

Hardy-Weinberg equilibrium (HW-E, p<0.05 was considered significant) was assessed using the chi-squared test. The I^2^ statistic was used to quantify the inconsistency between study estimates, and the Q statistic were used to formally test for heterogeneity (p<0.10 was considered representative of significant statistical heterogeneity). In this meta-analysis, a random-effects model was applied irrespective of between-study heterogeneity (DerSimonian & Laird). The association between Apo E polymorphism and POAG was estimated by calculating pooled odd ratios (ORs) and 95% CIs. The significance of the pooled OR was determined by Z test (P<0.05 was considered statistically significant). For allelic analysis, we examined the risk of POAG associated with ε2 and ε4 allele using ε3 as the reference group. For genotypic analysis, we defined ε3/ε3 genotype as the reference group. The ε2 carriers were defined as patients with the ε2/ε2 or ε2/ε3 genotype. The ε4 carriers included patients with the ε3/ε4 and ε4/ε4 genotype. The ε2 and ε4 carriers were separately compared with the ε3/ε3 group. (The ε2/ε4 genotype was excluded in genotypic analysis). All statistical analyses were carried out by using the Stata 12.0 (Stata Corporation, College Station, TX, USA).

### Sensitivity analysis

Subgroup analysis was used to investigate which factors (ethnicities, sources of controls, genotyping methods, HW-E or not, types of POAG) might contribute to the heterogeneity. One-way sensitivity analyses were performed by iteratively removing one study at a time to assess the stability of the meta-analysis results. Cumulative meta-analysis was performed to evaluate the accumulation of evidence on the association between Apo E polymorphisms and POAG.

### Publication bias

Publication bias was assessed using Begg's funnel plots and Egger's test. An asymmetric plot suggests a possible publication bias and the P value of Egger's test less than 0.05 was considered representative of statistically significant publication bias.

## Results

### Literature Search and Characteristics

**Figure 1:**
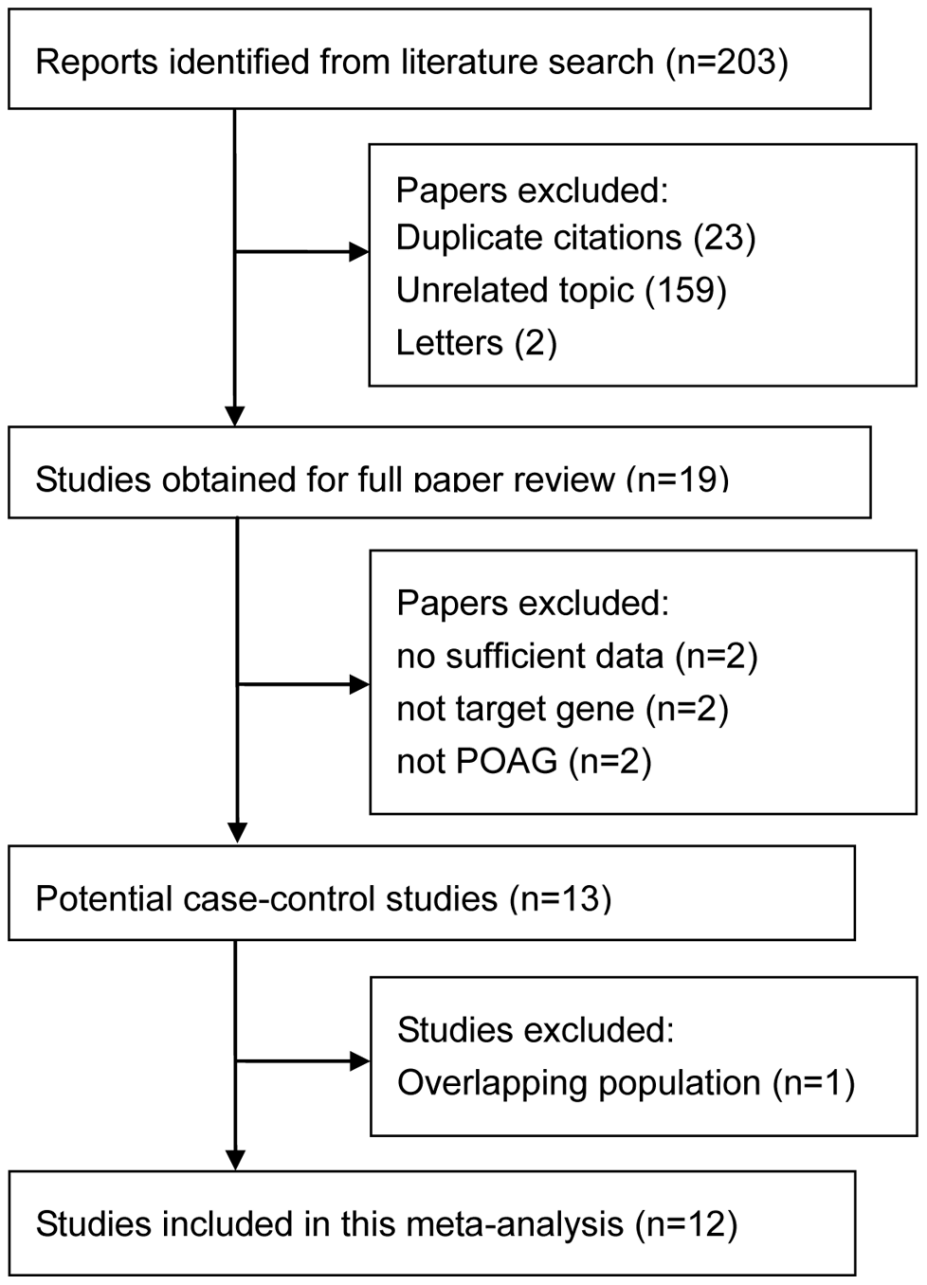
Flow diagram of included studies for this meta-analysis.

The study selection process is detailed in Fig. 1. The initial search strategy identified 203 studies. 184 were excluded (23 were duplicate studies, 159 were unrelated topic, two were letters), leaving 19 studies for full publication review. Of these, one article which contained overlapping data from the same patient source, 2 articles which absence of sufficient data for estimating OR and 95%CI, two article which were not about Apo E ε2/ε3/ε4 gene, two article which were not about POAG, were excluded. Thus, 12 studies were included in the final meta-analysis, including 1916 cases and 1756 controls.

All studies were case–control in design. Table 1 shows the studies identified and their main characteristics. Among these studies, two studies were not in HW-E, one study was unavailable for performing HW-E test. The NOS results showed that the average score was 7.42 (range 7 to 9), indicating that the methodological quality was generally good. There were 7 studies of Caucasian and 5 studies of Asian. Controls were mainly healthy populations and non-glaucoma participants. Five studies were population-based and 7 were hospital-based. The cases of 2 studies were patients with NTG, 3 studies were patients with HTG. Seven studies were mixed patients, among of them, 3 studies provided data concerning HTG and 2 studies had enough data for NTG, allowing subtype specific meta-analysis. Therefore, six studies were combined for HTG subtype and four for NTG subtype. MOOSE checklist was generated to provide detailed description of this meta-analysis (Table S1). Genotype and allele distributions for each case-control study are shown in Table S2.

**Table 1:** Characteristics of eligible studies included in the present meta-analysis.

First author(year)	Country	Ethnicity	Design		Patients			Control			
				Genotyping method	NO	Sex(M/F)	Age, y	NO	Sex(M/F)	Age, y	NOS score
Vickers(2002)	Australia	Caucasian	PB	PCR	142	NA	74.32±9.70	51	NA	83.2±7.0	9
Junemann(2004)	Germany	Caucasian	PB	NA	41	20/21	56.3	32	15/17	54.8	7
Lake(2004)	UK	Caucasian	PB	PCR-RFLP	155	65/90	70	349	150/199	55	7
Ressiniotis(2004)	UK	Caucasian	PB	Taqman assay	137	NA	73.0±8.0	75	NA	78.0±4.4	9
Mabuchi(2005)	Japan	Asian	HB	PCR-RFLP	310	156/154	63.5±14.4	179	62/117	65.5±11.6	7
Lam(2006)	China	Asian	HB	PCR-RFLP	400	250/150	61.0±17.2	300	191/109	70.4±9.3	7
Yuan(2007)	China	Asian	PB	PCR-RFLP	36	17/19	54.25±13.57	57	23/34	56.28±18.30	8
Zetterberg(2007)	Sweden	Caucasian	HB	minisequencing technique	242	187/155	70.6±9.2	187	51/136	65.8±6.9	7
Hu(2007)	China	Asian	HB	PCR-RFLP	142	NA	NA	77	NA	NA	7
Al-Dabbagh(2009)	Saudi	Caucasian	HB	PCR-RFLP	60	NA	58±14.4	130	102/28	45±11.6	7
Jia(2009)	China	Asian	HB	PCR	176	138/38	38.92±16.33	200	150/50	69.41±5.97	7
Saglar)2009)	Turkey	Caucasian	HB	PCR-RFLP	75	49/26	63.8±9.5	119	67/52	61.8±10.2	7

PB: population based; HB: hospital based; NA indicates data not available, PCR: polymerase chain reaction; RFLP restriction fragment length polymorphisms, M/F: male/female; NOS: Newcastle-Ottawa Scale.

### Main results and subgroup analyses

#### Allelic analysis

Table 2 give the summary results for the association of the Apo E polymorphism with the risk of POAG based on allelic analysis. The overall random effects pooled OR of ε2 versus ε3 for POAG showed no statistical significance: OR = 0.98 (95% CI: 0.79–1.23, P(Z) = 0.0872) (shown in Figure S1). Modest heterogeneity was present among the 11 studies (I^2^ = 30.6%, P(Q) = 0.155). For ε4 versus ε3, the results were also not statistically significant (shown in Figure S2). The summary OR was 1.05 (95% CI: 0.78–1.41; P(Z) = 0.743). The I^2^ statistic indicated substantial between-study heterogeneity (I^2^ = 70%, P(Q)<0.001).

**Table 2:** Summary estimates for the OR of Apo E porlymorphism in various allele/genotype contrasts: overall analysis and subgroup analyses.

			Test of association			Test of heterogeneity					overall test				
		Studies	OR	95%CI		Q	P(Q)^a^		I^2^		Z				P(Z)^b^
**Allelic analysis: ε2 allele vs ε3 allele**															
Overall		11	0.98	0.79	1.23	14.42	0.155	30.60%		0.16				0.872	
Ethnicity	Caucasian	6	1.05	0.75	1.45	7.93	0.160	36.90%		0.27				0.784	
	Asian	5	0.90	0.65	1.26	6.16	0.188	35.10%		0.60				0.551	
Source of controls	PB	5	0.88	0.60	1.28	5.55	0.236	27.90%		0.68				0.497	
	HB	6	1.04	0.78	1.40	8.32	0.139	39.90%		0.28				0.780	
Genotyping Method	PCR-RFLP	6	0.98	0.79	1.23	9.24	0.100	45.90%		0.07				0.942	
	Others	5	0.99	0.69	1.41	5.14	0.274	22.10%		0.19				0.846	
HW-E	Yes	8	1.02	0.80	1.29	10.33	0.171	32.20%		0.15				0.884	
	No	3	0.77	0.39	1.54	3.60	0.166	44.40%		0.74				0.458	
Type of POAG	HTG	6	0.81	0.52	1.26	11.30	0.046	55.70%		0.93				0.352	
	NTG	4	1.00	0.72	1.37	1.52	0.677	0.00%		0.03				0.975	
**Allelic analysis: ε4 allele vs ε3 allele**															
Overall		12	1.05	0.78	1.41	36.68	<0.001	70.00%		0.33				0.743	
Ethnicity	Caucasian	7	1.07	0.76	1.51	13.88	0.031	56.80%		0.37				0.709	
	Asian	5	1.06	0.61	1.82	21.62	<0.001	81.50%		0.20				0.844	
Source of controls	PB	5	1.18	0.71	1.94	13.29	0.010	69.90%		0.64				0.524	
	HB	7	0.97	0.67	1.40	19.71	0.003	69.60%		0.17				0.868	
Genotyping Method	PCR-RFLP	7	1.11	0.71	1.75	28.20	<0.001	78.70%		0.46				0.643	
	Others	5	1.01	0.70	1.48	8.25	0.083	51.50%		0.07				0.943	
HW-E	Yes	9	1.03	0.76	1.39	23.09	0.003	65.40%		0.19				0.851	
	No	3	1.03	0.36	2.96	12.71	0.002	84.30%		0.05				0.961	
Type of POAG	HTG	6	1.09	0.67	1.78	17.74	0.003	71.80%		0.36				0.715	
	NTG	4	1.07	0.59	1.93	10.24	0.017	70.70%		0.21				0.832	
**Genotypic analysis: ε2 carrier vs ε3/ε3**															
Overall		10	0.91	0.66	1.25	16.35	0.060	45.00%		0.61			0.543		
Ethnicity	Caucasian	5	1.04	0.69	1.55	5.36	0.252	25.40%		0.17			0.867		
	Asian	5	0.76	0.44	1.32	10.67	0.031	62.50%		0.97			0.331		
Source of controls	PB	4	0.77	0.48	1.24	2.52	0.472	0.00%		1.07			0.287		
	HB	6	0.97	0.64	1.46	12.75	0.026	60.80%		0.15			0.883		
Genotyping Method	PCR-RFLP	6	0.87	0.51	1.48	13.69	0.018	63.50%		0.53			0.599		
	Others	4	0.97	0.69	1.37	2.66	0.447	0.00%		0.15			0.879		
HW-E	Yes	8	0.91	0.65	1.29	15.20	0.033	54.00%		0.52			0.604		
	No	2	0.72	0.18	2.91	1.03	0.310	3.20%		0.47			0.640		
Type of POAG	HTG	6	0.84	0.49	1.42	9.72	0.083	48.60%		0.65			0.513		
	NTG	4	0.89	0.61	1.31	0.56	0.906	0.00%		0.58			0.564		
**Genotypic analysis: ε4 carrier carrier vs ε3/ε3**															
Overall		11	1.08	0.74	1.57	35.15	<0.001	71.60%		0.39		0.694			
Ethnicity	Caucasian	6	1.11	0.73	1.69	9.65	0.086	48.20%		0.50		0.620			
	Asian	5	1.08	0.56	2.11	23.84	<0.001	83.20%		0.23		0.820			
Source of controls	PB	4	1.62	0.62	4.28	15.11	0.002	80.10%		0.98		0.327			
	HB	7	0.90	0.62	1.30	15.97	0.014	62.40%		0.58		0.561			
Genotyping Method	PCR-RFLP	7	1.04	0.63	1.74	26.08	<0.001	77.00%		0.16		0.872			
	Others	4	1.19	0.70	2.02	6.44	0.092	53.40%		0.65		0.518			
HW-E	Yes	9	0.99	0.70	1.39	22.28	0.004	64.10%		0.07		0.946			
	No	2	1.65	0.14	19.81	10.03	0.002	90.00%		0.40		0.692			
Type of POAG	HTG	6	1.32	0.73	2.39	17.44	0.004	71.30%		0.93		0.353			
	NTG	4	1.34	0.52	3.44	21.06	<0.001	85.80%		0.60		0.549			

ε2 carriers indicates ε2/ε2+ε2/ε3 genotypes and ε4 carriers indicates ε4/ε4+ε3/ε4 genotypes. Apo E, apolipoprotein E; Q, Q-statistic; OR, odds ratio; CI, confidence interval; HW-E: Hardy–Weinberg equilibrium; PB: population based; HB: hospital based; HTG: hypertension glaucoma; NTG: normal tension glaucoma; PCR: polymerase chain reaction; RFLP restriction fragment length polymorphisms.

a Cochran's chi-square *Q* statistic test used to assess the heterogeneity in subgroups.

b *Z* test used to determine the significance of the overall OR.

Considering the fact that ethnic differences, sources of the controls, fulfilling HW-E or not, genotyping method, or type of POAG might bias the overall association, we conducted separate analysis according to these factors. The pooled OR for ε2 allele and ε4 allele versus ε3 allele were also not statistical significant in all subgroups.

#### Genotypic analysis

Table 2 show the summary results for the association between the Apo E genotype and POAG. The pooled OR for ε2 carriers and ε4 carriers versus ε3 carriers under the random effect model were 0.91 (95% CI: 0.66–1.25; P(Z) = 0.543) and 1.08 (95% CI: 0.74–1.57; P(Z) = 0.694), respectively (shown in Figure S3 and Figure S4). There was a substantial heterogeneity in both comparisons (ε2 carriers versus ε3 carriers: I^2^ = 45%, P(Q) = 0.060; ε4 carriers versus ε3 carriers: I^2^ = 71.6%, P(Q)<0.001). There was no material change in OR for both polymorphisms in subgroup analysis. Moreover, subgroup analysis also revealed significant heterogeneity for most comparisons.

#### Sensitivity analysis and cumulative meta-analysis

After the deletion of any single study, the random-effect estimates were not changed substantially, suggesting a high stability of the meta-analysis results. A cumulative meta-analysis based on sample size showed that the pooled OR remained centered on 1 with increasing sample size, indicating that Apo E ε2 and ε4 were unlikely risk variants for POAG (The data is not shown but is available on request.).

#### Publication Bias

Publication bias were qualitatively assessed by Begg's funnel plot and quantitatively assessed by Egger's test. Neither Begg's funnel plot nor Egger's test detected obvious evidence of publication bias n relation to allele or genotype. (shown in Figure S5).

## Discussion

The pathogenesis of POAG is complex and genetic factor play a important role in POAG susceptibility. An increasing number of articles on genetic association studies, genome-wide association studies (GWASs), and relate meta-analyses have been published to clarify the association between gene polymorphisms and POAG [23], [24]. To the authors' knowledge, this is the first meta-analysis investigating the association between Apo E ε2/ε3/ε4 polymorphisms and POAG. However, no significant association was found between Apo E polymorphisms and POAG after merging the results from 12 case-control studies. In subgroup analysis, associations between Apo E polymorphisms and POAG were also negative, although the number of articles included in this meta-analysis was limited.

Apo E is the major apolipoprotein of the central nervous system, where it is synthesized by glia, macrophages, and neurons [25]. In the rat eye, Apo E has been demonstrated to be synthesised by Muller cells, and secreted into the vitreous, where lipoproteins are assembled [26]. Apo E is absorbed by the ganglion cells (RGC), transported down the optic nerve, and may have a role in axonal nutrition [27]. There is a considerable body of evidence to show that Apo E genotype affects vulnerability of neurons to ischemia, survival and recovery after head injury, as well as its role in Alzheimer's disease [28]. Possession of the ε4 allele was shown to be associated with a reduced outcome after traumatic head injury and increased risk of earlier development of Alzheimer's disease [29]. Several case-control studies have investigated the association between Apo E polymorphism and risks of POAG. Some of them showed positive results, while others found no association. There are also some studies demonstrated that common polymorphisms in MYOC, OPTN, and Apo E might interactively contribute to POAG. Fan et al [18] observed that Apo E ε2/ε3/ε4 interact with OPTN Arg545Gln and MYOC -83G/A. Jia et al [15] identified another interactions between MYOC -83G>A and Apo E ε2/ε3/ε4. However, neither allele frequency nor genotype distribution was significantly associated with susceptibility to POAG in this meta-anlysis.

Heterogeneity is a potential problem that may affect the interpretation of the results. In our meta-analysis, significant heterogeneity was detected in some comparisons. To eliminate heterogeneity, we carried out subgroup analysis and used a random-effects model to pool the results whenever significant heterogeneity was present. Although the publication bias was maximally avoided, presence of between-study heterogeneity could not be fully explained by our subgroup analysis. It is unclear what factors contribute to the conflicting results reported in these studies. We speculate that several factors account for heterogeneity. Firstly, the diversity in the population characteristics may account for it. Different populations have different genetic backgrounds, which contribute to genetic heterogeneity. Secondly, environmental exposures and diet might play roles in these differences as well [30], [31]. In addition, some unpublished, eligible publications were not available in the present meta-analysis, which might affect the results. Thus, the results should be considered with caution, and in the future, more studies should be performed to assess these results.

Genome-wide association studies (GWAS) are a powerful tool for the identification of genetic risk factors for complex disease. POAG genetics has been the subject of several large scale GWAS in the past several years, and none has implicated Apo E [32], [33], [34], [35], [36], [37], [38]. Consistent with these studies, despite being an attractive candidate gene, our meta-analysis results do not support Apo E ε2/ε3/ε4 to have a major effect to POAG susceptibility.

However, caution should be made when interpreting the results due to some limitations of this study. Firstly, POAG is a multi-factorial disease that results from complex interactions between various genetic and environmental factors. Our results were based on unadjusted estimates, data were not stratified by other factors such as gender status, major systemic illness and family history, because sufficient information could not be extracted from the original studies. Secondly, this meta-analysis was limited by the number of cases and controls as well as small sample size, especially in subgroup analysis. However, given the available sample sizes and data, the study had 79% power to show a statistically association. This suggests that increasing the sample size would not change current results. All the included studies were carried out in Asians and Caucasians. Thus, the results may be applicable to these ethnic populations only. Thirdly, controls were not uniformly defined. This study is a meta-analysis of case-control studies, only 5 were population-based. Thus, some inevitable selection bias might exist in the results, and they may not be representative of the general population. Fouthly, all included studies were case-control design, which precludes further comments on cause-effect relationship. The results of long-term prospective, designed for the investigation of gene–gene and gene–environment interactions, in different ethnicity subgroups might produce more conclusive claims about the association between Apo E and POAG.

Despite of these limitations, this study also has some advantages. First, it provides pooled data on a substantial number of cases and controls for better understanding the association between Apo E polymorphism and POAG. In addition, the methodological issues for meta-analysis, such as, heterogeneity, publication bias, and stability of results were all well investigated.

In conclusion, despite the limitation, this meta-analysis suggests that Apo E ε2/ε3/ε4 polymorphisms may not be associated with the risk of POAG. However, to reach a definitive conclusion, well-designed studies with larger sample size and more ethnic groups should be considered to further clarify the association. Moreover, gene-gene and gene-environment interactions studies should also be considered in future studies.

## Supporting Information

Figure S1 **Forest plot for alleles of apolipoprotein E (Apo E) polymorphism and POAG risk in the overall study(ε2 allele vs ε3 allele).**
(TIF)

Figure S2 **Forest plot for alleles of apolipoprotein E (Apo E) polymorphism and POAG risk in the overall study(ε4 allele vs ε3 allele).**
(TIF)

Figure S3 **Forest plot for genotypes of apolipoprotein E (Apo E) polymorphism and POAG risk (ε2 carrier vs ε3/ε3).**
(TIF)

Figure S4 **Forest plot for genotypes of apolipoprotein E (Apo E) polymorphism and POAG risk (ε4 carrier vs ε3/ε3).**
(TIF)

Figure S5 **Begg's funnel plots of publication bias analyses**.
(DOC)

Table S1 **MOOSE checklist**.
(DOC)

Table S2 **The distribution of the ApoE genotypes and allele frequencies for cases and controls**.
(DOC)
