# Supplementary figures and images for: Lack of Association of Apolipoprotein E (Apo E) ε2/ε3/ε4 Polymorphisms with Primary Open-Angle Glaucoma: A Meta-Analysis from 1916 Cases and 1756 Controls
Source: PLoS One. 2013 Sep 2;8(9):e72644. doi: 10.1371/journal.pone.0072644 (PMC3759379; doi:10.1371/journal.pone.0072644)

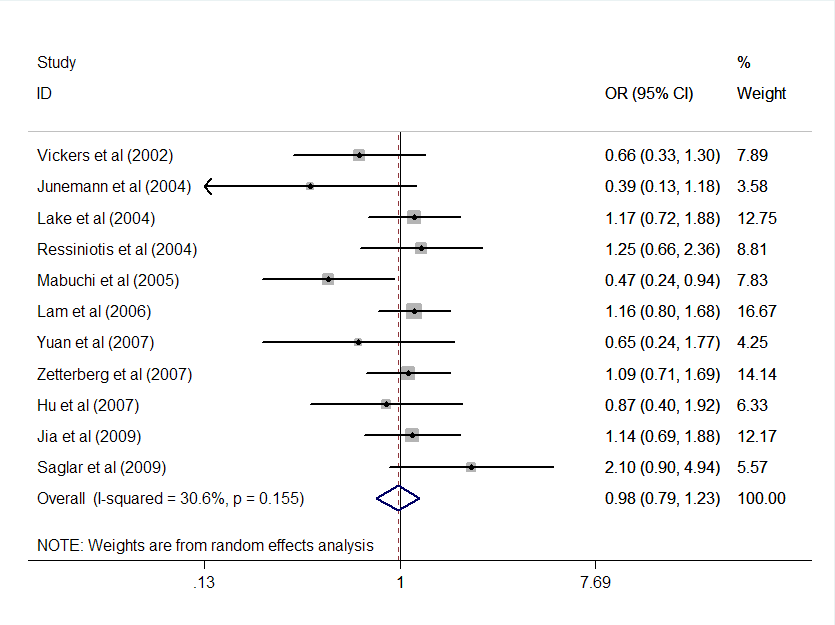

Supplement: Figure S1 — Forest plot for alleles of apolipoprotein E (Apo E) polymorphism and POAG risk in the overall study(ε2 allele vs ε3 allele). (TIF) [file pone.0072644.s001.tif]

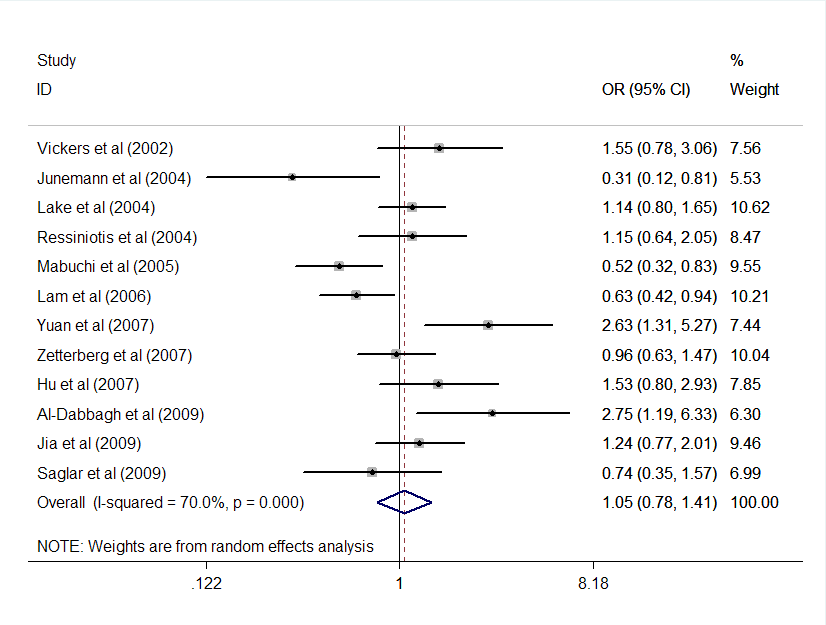

Supplement: Figure S2 — Forest plot for alleles of apolipoprotein E (Apo E) polymorphism and POAG risk in the overall study(ε4 allele vs ε3 allele). (TIF) [file pone.0072644.s002.tif]

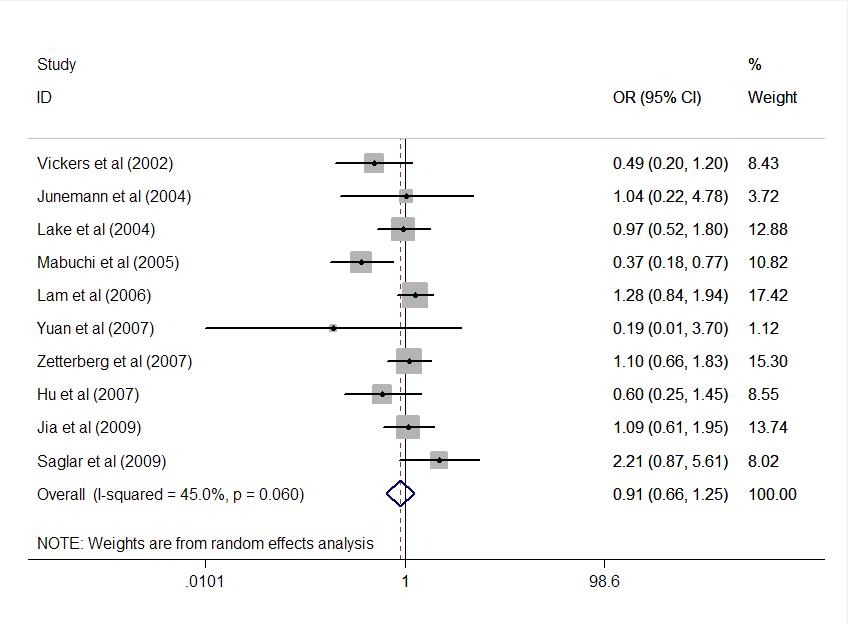

Supplement: Figure S3 — Forest plot for genotypes of apolipoprotein E (Apo E) polymorphism and POAG risk (ε2 carrier vs ε3/ε3). (TIF) [file pone.0072644.s003.tif]

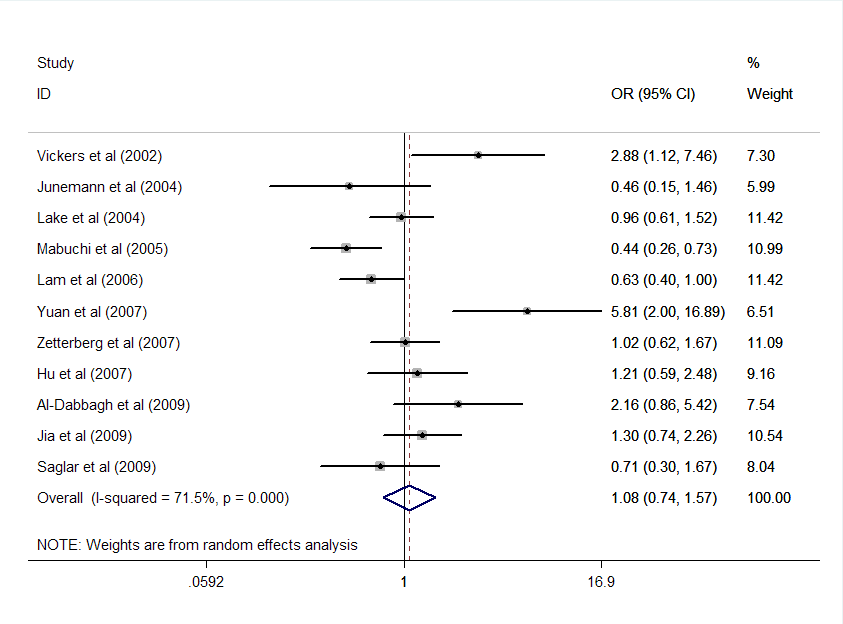

Supplement: Figure S4 — Forest plot for genotypes of apolipoprotein E (Apo E) polymorphism and POAG risk (ε4 carrier vs ε3/ε3). (TIF) [file pone.0072644.s004.tif]
