# Supplementary material for: Lack of Association of Apolipoprotein E (Apo E) ε2/ε3/ε4 Polymorphisms with Primary Open-Angle Glaucoma: A Meta-Analysis from 1916 Cases and 1756 Controls
Source: PLoS One. 2013 Sep 2;8(9):e72644. doi: 10.1371/journal.pone.0072644 (PMC3759379; doi:10.1371/journal.pone.0072644)
Supplement: Figure S5 — Begg's funnel plots of publication bias analyses. (DOC) [file pone.0072644.s005.doc]

**Supplementary Figure 1.** Egger’s funnel plots of publication bias analyses

ε2 allele versus ε3 allele

Egger test *P*=0.135

ε4 allele versus ε3 allele

Egger test *P*=0.449

ε2 carriers versus ε3 carriers

Egger test *P*=0.224

ε4 carriers versus ε3 carriers

Egger test *P*=0.103
