# Supplementary material for: Lack of Association of Apolipoprotein E (Apo E) ε2/ε3/ε4 Polymorphisms with Primary Open-Angle Glaucoma: A Meta-Analysis from 1916 Cases and 1756 Controls
Source: PLoS One. 2013 Sep 2;8(9):e72644. doi: 10.1371/journal.pone.0072644 (PMC3759379; doi:10.1371/journal.pone.0072644)
Supplement: Table S1 — MOOSE checklist. (DOC) [file pone.0072644.s006.doc]

**MOOSE Checklist**

| **Criteria** | | **Brief description of how the criteria were handled in the meta-analysis** | **Page** |
| --- | --- | --- | --- |
| **Reporting of background should include** | | |  |
| √ | Problem definition | Apolipoprotein E(Apo E) play crucial roles in various biological processes and may be associated with the risk for primary open-angle glaucoma (POAG). The association between Apo E ε2/ε3/ε4 polymorphisms and susceptibility to POAG were inconsistent in previous studies. | 3 |
| √ | Hypothesis statement | Apo E ε2/ε3/ε4 polymorphisms may affect susceptibility to POAG. | 3,4 |
| √ | Description of study outcomes | Primary open-angle glaucoma | 4,5 |
| √ | Type of exposure or intervention used | ε2 carriers (ε2/ε3+ε2/ε2) genotypes or ɛ2 allele in Apo E  ε4 carriers (ε3/ε4+ε4/ε4) genotypes or ε4 allele in Apo E | 6 |
| √ | Type of study designs used | Case-control studies. | 5 |
| √ | Study population | No restriction | 5 |
| **Reporting of search strategy should include** | | |  |
| √ | Qualifications of searchers | Investigators include experts in glaucoma, biologists and qualified graduate students. All of the investigators have received training in literature research, statistics and evidence-based medicine. | 4 |
| √ | Search strategy, including time period included in the synthesis and keywords | Publication time was not restricted. We searched all available literatures in the following databases: Pubmed, Embase, ISI Wed of Knowledge, Cochrane Library and Chinese databases such as the China National Knowledge Infrastructure (CNKI) and Wanfang  Key words: apolipoprotein E or Apo E, polymorphism (s) or allele (s) variation or genotype (s) and glaucoma or intraocular hypertension | 4 |
| √ | Databases and registries searched | Pubmed, Embase, ISI Wed of Knowledge, Cochrane Library and Chinese databases such as the China National Knowledge Infrastructure (CNKI) and Wanfang | 4,5 |
| √ | Search software used, name and version, including special features | We did not employ any search software. EndNote was used to merge retrieved citations and eliminate duplications. | 4,5 |
|  | Use of hand searching | Reference lists of retrieved fulltexts were searched manually | 5 |
| √ | List of citations located and those excluded, including justifications | Literature search and selection process are outlined in the flow diagram. The reasons for exclusion were listed in the flow diagram. The citation list is available upon request. | 7 |
|  | Method of addressing articles published in languages other than English | Language was not restricted. | 4 |
| √ | Method of handling abstracts and unpublished studies | We first examined if overlap existed and excluded overlapped studies. We tried to contact authors for unpublished data. If authors were not willing to provide data or the requests were not responded, we excluded the study. | 4 |
| √ | Description of any contact with authors | We contacted researchers who conducted related studies to obtain detailed genotype frequencies. However, none of the researchers agreed to provide the data we needed. | 5 |
| **Reporting of methods should include** | | |  |
| √ | Description of relevance or appropriateness of studies assembled for assessing the hypothesis to be tested | Detailed inclusion and exclusion criteria were described in the methods. | 5 |
| √ | Rationale for the selection and coding of data | Data extracted from each of the studies were relevant to the population characteristics, study design, genotyping methods, genotypes, outcome, and possible effect modifiers of the association. | 5 |
| √ | Assessment of confounding | Subgroup analysis in ethnicities, sources of controls，genotyping methods, HW-E or not, types of POAG were performed and we conducted sensitivity analysis by deleting a single study one by one for each time. We perform cumulative meta-analysis by sample size. | 6 |
| √ | Assessment of study quality, including blinding of quality assessors; stratification or regression on possible predictors of study results | We assessed the methodological qualities of included studies by the Newcastle-Ottawa Scale. | 5 |
| √ | Assessment of heterogeneity | Statistical heterogeneity among the studies was checked by chi-square-based *Q*-test. A *P*-value greater than 0.10 for *Q*-test indicates no significant heterogeneity existed among studies | 6 |
| √ | Description of statistical methods in sufficient detail to be replicated | Methods of heterogeneity test, quantitative synthesis, assessments of publication bias, sensitivity analyses are reported in detail in the methods section. | 6 |
| √ | Provision of appropriate tables and graphics | We provided flow chart to explain literature searching and selection; the explanation of sensitivity analysis, study characteristics and allele/genotype frequencies; pooled analysis of Apo E. | 16-19 |
| **Reporting of results should include** | | |  |
|  | Graph summarizing individual study estimates and overall estimate | We did not provide individual forest plot. Individual pooled ORs, 95% CIs and *P*-values for *Z* test were summarized in Table 2. | 7,8 |
| √ | Table giving descriptive information for each study included | Descriptive information for each study included was provided in Table 1. | 7 |
| √ | Results of sensitivity testing | The results of subgroup and sensitivity analysis were described in results section (Table 2) | 8 |
| √ | Indication of statistical uncertainty of findings | The results of heterogeneity test, pooled ORs, 95% confidence intervals and *P* value for *Z* test were presented with all pooled analyses. Results of sensitivity analysis were described and explained. | 8 |
| **Reporting of discussion should include** | | |  |
| √ | Quantitative assessment of bias | We evaluated the publication bias by funnel plots and egger’s test. No significant publication bias was detected. | 8 |
| √ | Justification for exclusion | Only case-control studies which meet our inclusion criteria were included. Reviews, meta-analysis, studies on other glaucoma than POAG and other irrelevant studies which were inconsistent to the inclusion criteria were excluded. | 6 |
| √ | Assessment of quality of included studies | We discussed the results of sensitivity analysis and described the limitations of included studies. | 6,7 |
| **Reporting of conclusions should include** | | |  |
| √ | Consideration of alternative explanations for observed results | We discussed that potential unmeasured confounders and explained the limitations of this meta-analysis. We reminded readers that caution should be made when interpreting this meta-analysis. | 9,10 |
| √ | Generalization of the conclusions | This meta-analysis suggests that Apo E ε2/ε3/ε4 polymorphisms may not be associated with the risk of POAG. | 11 |
| √ | Guidelines for future research | Further well-designed studies with larger sample size and more ethnic groups are needed to further validate the association. | 11 |
| √ | Disclosure of funding source | This study was supported in whole or in part by the National Natural Science Foundation of China (81170849), Guangdong Provincial Natural Science Foundation (S2011020002401), and the Fundamental Research Funds of State Key Laboratory of Ophthalmology (2011C02). | 1 |
