# Supplementary material for: Lack of Association of Apolipoprotein E (Apo E) ε2/ε3/ε4 Polymorphisms with Primary Open-Angle Glaucoma: A Meta-Analysis from 1916 Cases and 1756 Controls
Source: PLoS One. 2013 Sep 2;8(9):e72644. doi: 10.1371/journal.pone.0072644 (PMC3759379; doi:10.1371/journal.pone.0072644)
Supplement: Table S2 — The distribution of the ApoE genotypes and allele frequencies for cases and controls. (DOC) [file pone.0072644.s007.doc]

**Supplementary Table 2.** The distribution of the ApoE genotypes and allele frequencies for cases and controls.

| Study included | Genotype for cases, n | | | | | |  | Genotype for control, n | | | | | |  | Allele for cases, n | | |  | Allele for controls, n | | |  | HW-E* |
| --- | --- | --- | --- | --- | --- | --- | --- | --- | --- | --- | --- | --- | --- | --- | --- | --- | --- | --- | --- | --- | --- | --- | --- |
|  | ε2/ε2 | ε2/ε3 | ε2/ε4 | ε3/ε3 | ε3/ε4 | ε4/ε4 |  | ε2/ε2 | ε2/ε3 | ε2/ε4 | ε3/ε3 | ε3/ε4 | ε4/ε4 |  | ε2 | ε3 | ε4 |  | ε2 | ε3 | ε4 |  | P-Value |
| Vickers(2002)[1] | 6 | 8 | 7 | 78 | 42 | 3 |  | 2 | 9 | 2 | 30 | 6 | 0 |  | 27 | 206 | 51 |  | 15 | 75 | 12 |  | 0.563 |
| Junemann(2004)[2] | 0 | 6 | 0 | 27 | 8 | 0 |  | 0 | 3 | 6 | 14 | 9 | 0 |  | 6 | 68 | 8 |  | 9 | 40 | 15 |  | 0.012 |
| Lake(2004)[3] | 1 | 16 | 10 | 91 | 31 | 6 |  | 3 | 37 | 13 | 208 | 81 | 7 |  | 28 | 229 | 53 |  | 56 | 534 | 108 |  | 0.316 |
| Ressiniotis(2004)[4] | NA | NA | NA | NA | NA | NA |  | NA | NA | NA | NA | NA | NA |  | 35 | 199 | 40 |  | 16 | 114 | 20 |  | NA |
| Mabuchi(2005)[5] | 0 | 14 | 2 | 259 | 35 | 0 |  | 0 | 18 | 0 | 123 | 38 | 0 |  | 16 | 567 | 37 |  | 18 | 302 | 38 |  | 0.104 |
| Lam(2006)[6] | 0 | 74 | 5 | 280 | 40 | 1 |  | 0 | 42 | 8 | 203 | 47 | 0 |  | 79 | 674 | 47 |  | 50 | 495 | 55 |  | 0.065 |
| Yuan(2007)[7] | 0 | 0 | 6 | 12 | 15 | 3 |  | 0 | 6 | 12 | 31 | 8 | 0 |  | 6 | 39 | 27 |  | 18 | 76 | 20 |  | <0.01 |
| Zetterberg(2007)[8] | 2 | 34 | 4 | 110 | 35 | 2 |  | 1 | 42 | 6 | 145 | 44 | 4 |  | 42 | 289 | 43 |  | 50 | 376 | 58 |  | 0.720 |
| Hu(2007)[9] | 1 | 11 | 4 | 95 | 28 | 3 |  | 0 | 11 | 0 | 52 | 14 | 0 |  | 17 | 229 | 38 |  | 11 | 129 | 14 |  | 0.409 |
| Al-Dabbagh(2009)[10] | 0 | 0 | 0 | 50 | 7 | 3 |  | 0 | 0 | 0 | 119 | 11 | 0 |  | 0 | 107 | 13 |  | 0 | 249 | 11 |  | 0.968 |
| Jia(2009)[11] | 2 | 25 | 5 | 112 | 29 | 3 |  | 1 | 29 | 4 | 136 | 28 | 2 |  | 34 | 280 | 38 |  | 35 | 329 | 36 |  | 0.897 |
| Saglar(2009)[12] | 0 | 12 | 1 | 53 | 8 | 1 |  | 0 | 9 | 1 | 88 | 19 | 2 |  | 13 | 126 | 11 |  | 10 | 204 | 24 |  | 0.832 |

HW-E, Hardy-Weinberg equilibrium; *X^2^-based statistic test for Hardy-Weinberg equilibrium among control subjects for each study (df=3). NA: no available

**References:**

1. Vickers JC, Craig JE, Stankovich J, McCormack GH, West AK, et al. (2002) The apolipoprotein epsilon4 gene is associated with elevated risk of normal tension glaucoma. Mol Vis 8: 389-393.

2. Junemann A, Bleich S, Reulbach U, Henkel K, Wakili N, et al. (2004) Prospective case control study on genetic assocation of apolipoprotein epsilon2 with intraocular pressure. Br J Ophthalmol 88: 581-582.

3. Lake S, Liverani E, Desai M, Casson R, James B, et al. (2004) Normal tension glaucoma is not associated with the common apolipoprotein E gene polymorphisms. Br J Ophthalmol 88: 491-493.

4. Ressiniotis T, Griffiths PG, Birch M, Keers S, Chinnery PF (2004) The role of apolipoprotein E gene polymorphisms in primary open-angle glaucoma. Arch Ophthalmol 122: 258-261.

5. Mabuchi F, Tang S, Ando D, Yamakita M, Wang J, et al. (2005) The apolipoprotein E gene polymorphism is associated with open angle glaucoma in the Japanese population. Mol Vis 11: 609-612.

6. Lam CY, Fan BJ, Wang DY, Tam PO, Yung TC, et al. (2006) Association of apolipoprotein E polymorphisms with normal tension glaucoma in a Chinese population. J Glaucoma 15: 218-222.

7. Yuan HP, Xiao Z, Yang BB (2007) A study on the association of apolipoprotein E genotypes with primary open-angle glaucoma and primary angle-closure glaucoma in northeast of China. Zhonghua Yan Ke Za Zhi 43: 416-420.

8. Zetterberg M, Tasa G, Palmer MS, Juronen E, Teesalu P, et al. (2007) Apolipoprotein E polymorphisms in patients with primary open-angle glaucoma. Am J Ophthalmol 143: 1059-1060.

9. Hu Y (2007) The APOE gene and its interactions with SNPs of other genes in primary open angle glaucoma and age-related macular degeneration [Master degree]. Shantou, China: Joint Shantou International Eye Center of Shantou University and Chinese University of Hongkong.

10. Al-Dabbagh NM, Al-Dohayan N, Arfin M, Tariq M (2009) Apolipoprotein E polymorphisms and primary glaucoma in Saudis. Mol Vis 15: 912-919.

11. Jia LY, Tam PO, Chiang SW, Ding N, Chen LJ, et al. (2009) Multiple gene polymorphisms analysis revealed a different profile of genetic polymorphisms of primary open-angle glaucoma in northern Chinese. Mol Vis 15: 89-98.

12. Saglar E, Yucel D, Bozkurt B, Ozgul RK, Irkec M, et al. (2009) Association of polymorphisms in APOE, p53, and p21 with primary open-angle glaucoma in Turkish patients. Mol Vis 15: 1270-1276.
